# A Sentinel-1 SAR imagery dataset for airstrips detection and segmentation in the Brazilian Amazon Rainforest

**DOI:** 10.1016/j.dib.2026.112472

**Published:** 2026-01-14

**Authors:** Leandro da Silva Gomes, Gustavo Henrique de Queiroz Stabile, Tahisa Neitzel Kuck, Felipe Augusto Pereira de Figueiredo, Elcio Hideiti Shiguemori, Dimas Irion Alves

**Affiliations:** a Aeronautics Institute of Technology (ITA): Praça Marechal Eduardo Gomes, 50, Vila das Acácias, São José dos Campos, SP 12228-900, Brazil; b Institute for Advanced Studies (IEAv): Trevo Coronel Aviador José Alberto Albano do Amarante, 1, Putim, São José dos Campos, SP 12228-001, Brazil; c Instituto Nacional de Telecomunicações (INATEL): Av. João de Camargo, 510, Centro, Santa Rita do Sapucaí, MG 37540-000, Brazil

**Keywords:** Deep learning, Object detection, Semantic segmentation, Geospatial dataset

## Abstract

The Brazilian Amazon Rainforest holds a large ecological and economic importance and is considered one of the most biodiverse regions on the planet. The region faces numerous challenges from illegal human activities that threaten its sustainability and well-being, which are often supported by the construction of unauthorized airstrips. Additionally, due to its persistent cloud cover, which often hinders monitoring with optical satellites, Synthetic Aperture Radar (SAR) imagery provides a crucial alternative for the region surveillance. Thus, this dataset was developed to support the training and evaluation of machine learning techniques, including deep learning models for detecting and segmenting airstrips in the Brazilian Amazon Rainforest using SAR imagery. The dataset comprises images from the Sentinel-1 satellite, acquired primarily between 2021 and 2024, covering 1040 locations of known airstrips sourced from the MapBiomas project (published in 2023, based on 2021 reference data). For the change detection task, historical “before” images were selected from the period between 2014 and 2021 to capture the pre-construction state. The data is structured to support three distinct machine learning tasks: object detection (e.g., YOLOv8), semantic segmentation (e.g., U-Net), and change detection. For each task, specific images and annotations are provided. Additionally, geospatial files (Shapefile, GeoPackage) are included to facilitate the integration and visualization of the dataset in a GIS environment. The data is valuable for researchers in remote sensing, computer vision, environmental monitoring, security and defense, enabling the development of automated systems to monitor irregular activities in remote forest regions. The dataset is available at a Mendeley Data repository: https://data.mendeley.com/datasets/x7rn78ymtn/1

Specifications Table

**Table:** 

Subject	Earth & Environmental Sciences
Specific subject area	Illegal activity monitoring, SAR image processing, Deep Learning, Object Detection, Semantic Segmentation, Change Detection.
Type of data	Images (GeoTIFF, PNG), Annotations (TXT), Binary Masks (GeoTIFF), Geospatial vector data (Shapefile, GeoPackage, CSV).
Data collection	Coordinates sourced from MapBiomas (v.2023). From 2869 catalogued airstrips, only the 1040 locations with available Sentinel-1 imagery were selected. Images acquired via Google Earth Engine API using a Python script on Google Colab. Instrument: Sentinel-1 C-band SAR (Interferometric Wide (IW) mode, Vertical-Vertical (VV) polarization, Ground Range Detected (GRD) product). Date range: 2021–2024 (main) and 2014–2021 (historical). Binary masks manually annotated in QGIS by a specialist, georeferenced over high-resolution Planet (NICFI) optical imagery.
Data source location	Brazilian Amazon Rainforest (1040 distinct locations). Geographic coordinates for each point are provided in the auxiliary data.
Data accessibility	Repository name: Mendeley Data Data identification number: DOI 10.17632/x7rn78ymtn.1 Direct URL to data: https://data.mendeley.com/datasets/x7rn78ymtn/1 [1]
Related research article	None.

## 1 Value of the Data

The provided dataset is valuable for the scientific community for several reasons:

• It directly addresses the monitoring of logistical infrastructure (clandestine airstrips) that supports illegal gold mining ("garimpo") and drug trafficking in the Amazon. These targets are particularly challenging to detect due to their narrow width (often <20 m), unpaved surfaces, and similarity to other linear features like rivers and roads.
• It enables the benchmarking of deep learning models specifically on Sentinel-1 C-band SAR imagery within a complex tropical forest environment. This allows researchers to test the robustness of algorithms against SAR speckle noise and geometric distortions in regions with persistent cloud cover.
• The dataset structure supports a complete monitoring pipeline through three complementary tasks: Object Detection (for rapid identification of potential targets), Semantic Segmentation (for estimating airstrip dimensions and usable length), and Change Detection (for identifying the construction of new airstrips or the expansion of existing ones).
• The inclusion of georeferenced auxiliary data (shapefiles and coordinates) allows for immediate integration with official land use databases, enabling researchers to cross-reference detections with indigenous lands and conservation units to assess environmental impact.
• To our knowledge, this is the first open-source labeled dataset focused on unauthorized airstrips in the Amazon context, filling a gap in remote sensing literature which predominantly focuses on ships, vehicles, or urban infrastructure.

## 2 Background

The Brazilian Amazon Rainforest is a vast, biodiverse region where illegal human activities, often supported by clandestine airstrips, threaten sustainability and well-being [2]. Systematic monitoring of these structures is crucial for combating illicit activities [3,4], but the region’s vastness and persistent cloud cover render field-based monitoring difficult and optical remote sensing ineffective [[5], [6], [7]]. Synthetic Aperture Radar (SAR) technology offers a vital all-weather, day-and-night monitoring alternative [6,8].

Once SAR images are acquired, the challenge shifts to processing the large data volume to identify airstrips [9]. This involves distinguishing small, often narrow linear structures (which can be <20 m wide) within heterogeneous landscapes. While AI and Convolutional Neural Networks (CNNs) are effective for SAR target detection [[10], [11], [12], [13], [14], [15]], research specifically on airstrips using C-band Sentinel-1 data is scarce [16,17]. This dataset was created as the foundation for research presented in [16] and [18], which evaluated deep learning models (YOLOv8 and U-Net) for airstrip detection, segmentation, and change detection. This dataset comprises the complete collection of images and labels generated for that work, providing a benchmark resource for automated monitoring systems in the Amazon.

## 3 Data Description

**Fig. 1:**
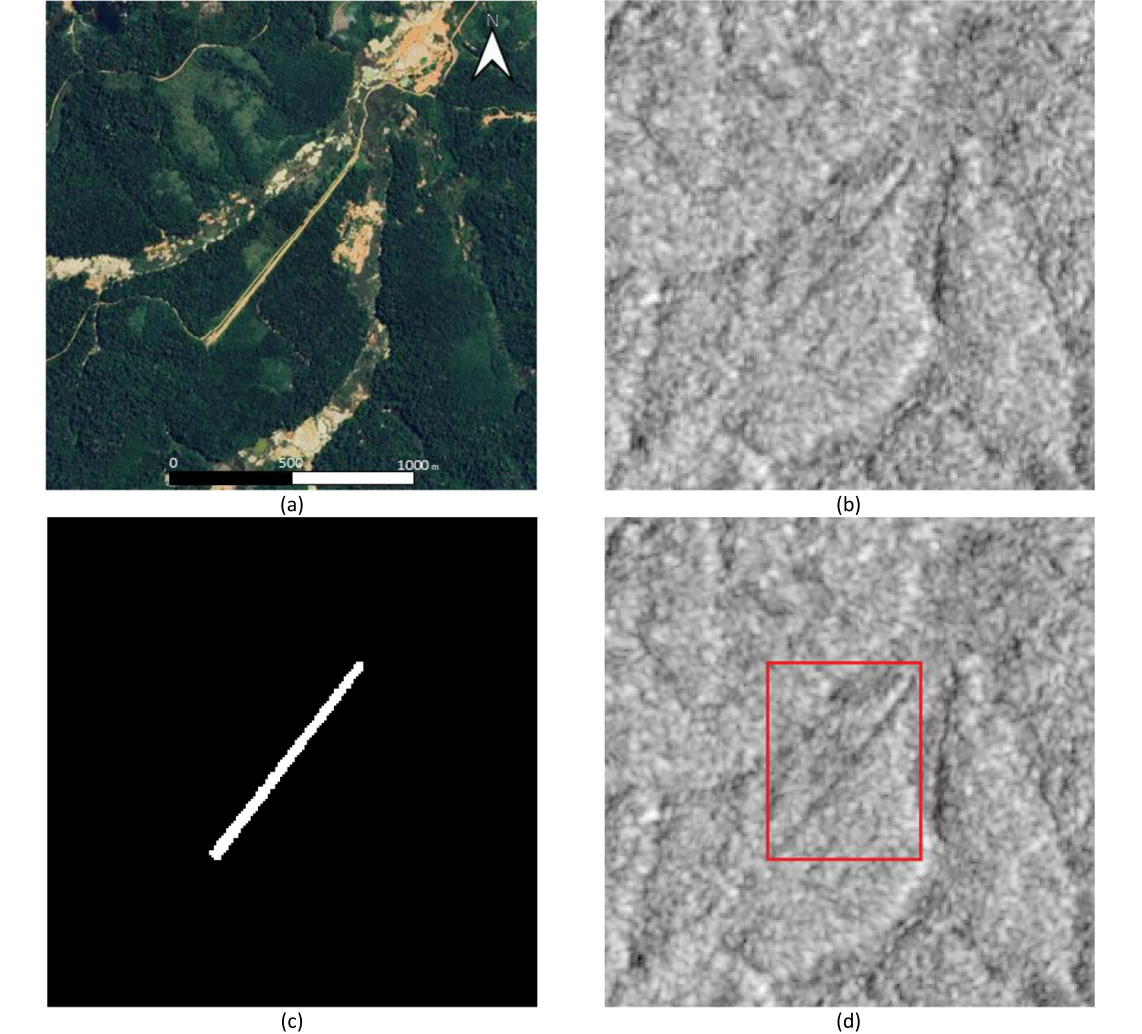
Data representation and annotation formats. (a) Example optical image (not in dataset). (b) Corresponding Sentinel-1 SARmimage. (c) Binary segmentation mask. (d) Bounding box annotation overlaid on the SAR image for the object detection task. Scale bar in (a) represents 1000 m and applies to all images. North is up.

**Fig. 2:**
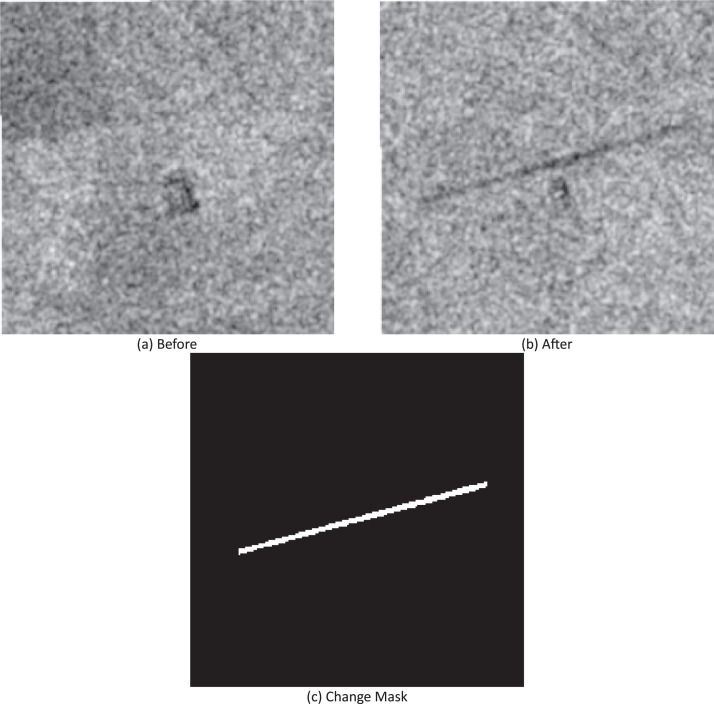
Example of data for the change detection task. (a) SAR image of the location before the airstrip’s construction. (b) SAR image of the same location after the airstrip’s construction. (c) Binary segmentation mask indicating the newly built airstrip.

The dataset is organized into a main directory containing several sub-folders with the images, annotations, and supplementary geospatial data. All files (images, masks, annotations) share a common ID for easy matching (e.g., “_ID_25.tif”, “_ID_25.png”, “_ID_25.txt”). A visual guide to the data formats for each task is provided in Fig. 1, Fig. 2.

• “Images_geotiff”: Contains 1040 core images in GeoTIFF format (200 × 200 pixels, 1-band, Float64). This folder represents the highest quality data source, preserving the original σ0 backscatter values, and serves as the primary input for segmentation and change detection tasks.
• “Images_png”: Contains 1040 images in PNG format (200 × 200 pixels, 8-bit). These are processed versions of the GeoTIFF images, optimized for object detection CNN frameworks like YOLO that often require 8-bit inputs.
• “Segmentation_masks”: Contains 1040 binary segmentation masks in GeoTIFF format (8-bit). Each mask is identified by ID and corresponds to an image in ‘images_geotiff’. Pixels representing the airstrip have a value of 255 (white), while the background has a value of 0 (black).
• “Bbox_annotations”: Contains 1040 “.txt” files with bounding box coordinates in the YOLO format (“class_id x_center y_center width height”). All targets belong to a single class (ID “0″). Each annotation file corresponds by ID to an image in “Images_png”.
• “Change_detection”: This folder contains data for 113 locations where a clear “before” and “after” scene was available.
  ○ “before”: GeoTIFF images captured before the airstrip’s construction.
  ○ “after”: GeoTIFF images captured after the airstrip’s construction. These files are duplicates of the corresponding images in the main “images_geotiff” folder, provided here for convenience.
  ○ “mask”: Binary masks corresponding to the change (the new airstrip) visible in the “after“ image.

• “Geodata”: Contains auxiliary geospatial files to facilitate analysis and visualization:
  ○ “airstrips_in_dataset”: A Shapefile with the location and geometry of the airstrips included in this dataset, as sourced from MapBiomas [19].
  ○ “airstrips_coordinates.csv”: A CSV file listing the unique ID and geographic coordinates (latitude, longitude) of all 1040 airstrips.
  ○ “images_contour.gpkg”: A GeoPackage file containing the georeferenced footprint of each image tile, allowing for easy visualization of the dataset’s spatial coverage in a GIS software.

## 4 Experimental Design, Materials and Methods

**Fig. 3:**
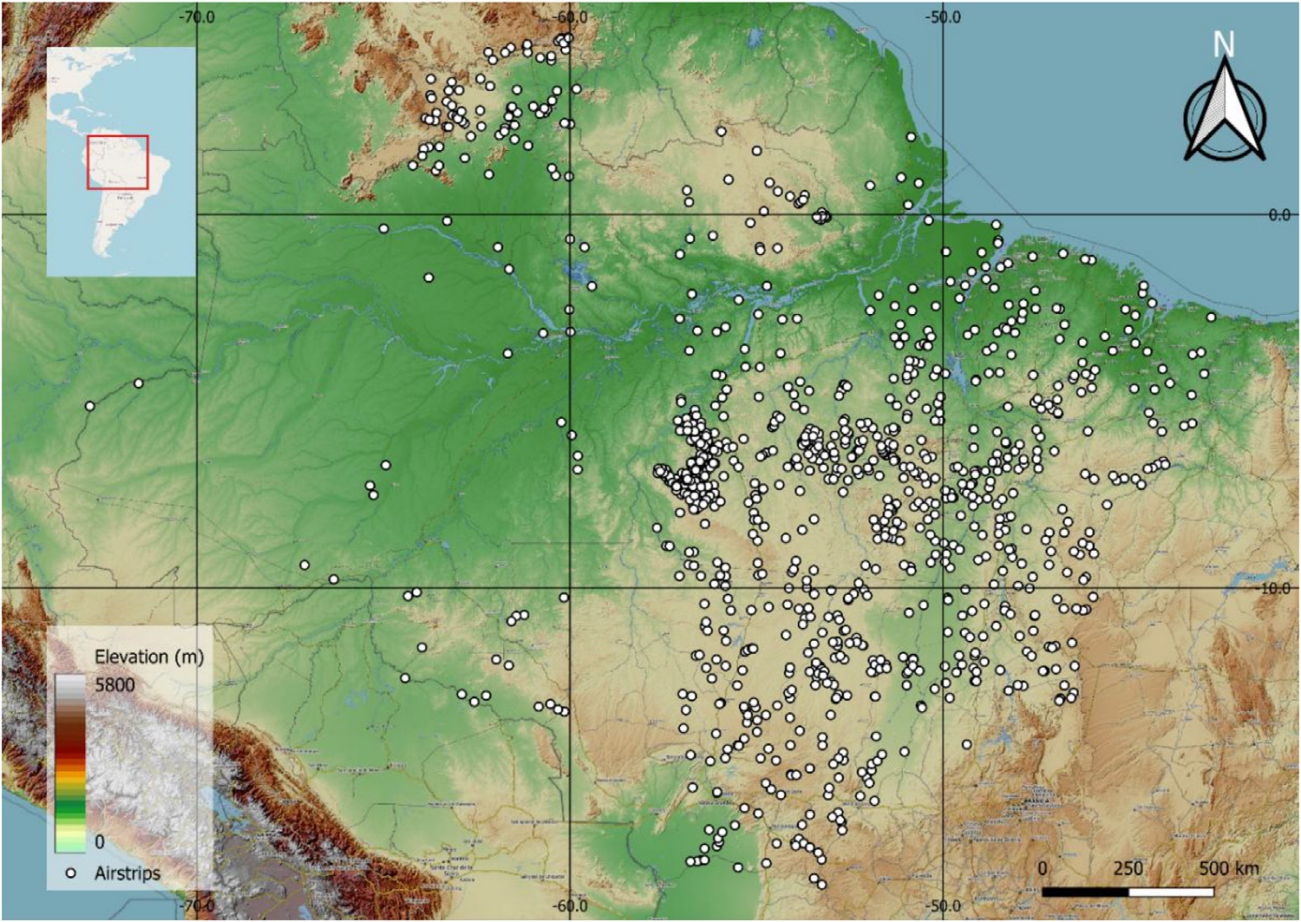
Geographic distribution of the 1040 airstrips available in the dataset.

The dataset has been generated following a systematic procedure. The locations of airstrips were obtained from the MapBiomas project’s 2023 database [19]. From the complete catalog provided by MapBiomas, Sentinel-1 SAR GRD images available on the Google Earth Engine (GEE) platform were identified for 1040 unique locations, which constitute this dataset. Fig. 3 shows the distribution of the airstrips over the Amazon Rainforest. A Python script was developed in the Google Colab environment to query the GEE API and download the imagery for these specific coordinates.

For each coordinate, a 200 × 200 pixel image tile, corresponding to a 4 km² area centered on the airstrip’s location, was extracted. This dimension was chosen to ensure that the entire target airstrip, generally shorter than 2 km in length, was fully contained within the image tile. The Sentinel-1 data used were in Interferometric Wide (IW) mode with VV polarization. The images were queried from GEE as analysis-ready data, as the standard Sentinel-1 GRD preprocessing pipeline was already applied on the platform. These preprocessing steps are necessary to ensure the images are radiometrically and geometrically corrected before their use in further analysis. This pipeline includes: (1) Orbit file application, (2) Border and thermal noise removal, (3) Radiometric calibration to σ0 (sigma-nought), and (4) Terrain correction (orthorectification) using the SRTM 30-meter DEM.

The data were then processed for the specific machine learning tasks. Pixel-wise binary segmentation masks (0 for background, 255 for the airstrip) were manually created by a specialist. To ensure clear target identification, this annotation was performed by referencing high-resolution optical images from the PLANET constellation (used as ground truth) rather than annotating the SAR images directly. For the object detection task, these segmentation masks were then programmatically converted into bounding box coordinates in the YOLO format using a custom Python script, ensuring consistency between the two annotation types. The Float64 GeoTIFFs were also converted to 8-bit PNGs specifically for the object detection task, as this format is optimized for object detection frameworks like YOLO that often require 8-bit inputs. For change detection, a temporal query was performed to find “before” and “after” image pairs. This process yielded only 113 locations, as it was particularly difficult to source temporally-appropriate images that clearly captured the construction period of the airstrips. A corresponding mask (created as described above) for the “after” image was included for these 113 pairs.

## Limitations

A primary limitation of this dataset is the inherent difficulty of data acquisition. The 1040 locations represent a subset of the 2869 airstrips cataloged by MapBiomas [19]. This reduction is due to the challenge of finding corresponding usable Sentinel-1 images in the Google Earth Engine archive for every known coordinate. Furthermore, as noted in the methodology, the change detection subset is limited to 113 pairs.

A secondary methodological limitation relates to the ground truth generation. As noted in the methodology, the segmentation masks were derived from optical images rather than direct SAR annotation. While this approach was necessary for clear target identification, it may introduce minor spatial misalignments between the optical-derived masks and the SAR image geometry, especially in areas with complex terrain.

Despite these challenges, the dataset’s unique value lies in its specificity. To our knowledge, it is the first publicly available, multi-task dataset (supporting object detection, segmentation, and change detection) for airstrip monitoring in the Amazon using C-band SAR imagery. The provision of data in multiple formats (Float64 GeoTIFF, 8-bit PNG) and rich geospatial files (Shapefile, GPKG, CSV) makes it a valuable, ready-to-use resource for benchmarking automated monitoring systems in this critical region.

## Ethics Statement

The authors confirm that, after reading the ethical requirements for publication in Data in Brief, the current work does not involve human subjects, animal experiments, or any data collected from social media platforms.

## CRediT author statement

**Leandro da Silva Gomes:** Conceptualization, Methodology, Software, Validation, Formal analysis, Investigation, Data Curation, Writing - Original Draft, Visualization. **Gustavo Henrique de Queiroz Stabile:** Software, Validation, Formal analysis, Data Curation, Writing - Original Draft, Visualization. **Tahisa Neitzel Kuck:** Supervision, Conceptualization, Methodology, Validation, Formal analysis, Data Curation, Writing - Review & Editing, Resources. **Felipe Augusto Pereira de Figueiredo:** Validation, Writing – Review &Editing, Resources, Funding acquisition. **Elcio Hideiti Shiguemori:** Supervision, Conceptualization, Methodology, Writing - Review & Editing, Resources. **Dimas Irion Alves:** Supervision, Conceptualization, Methodology, Writing - Review & Editing, Project administration, Funding acquisition

## Data Availability

Mendeley DataS1-AAD: Sentinel-1 Amazon Airstrip Dataset (Original data)
